# Supplementary material for: The Prognostic Value of the Hedgehog Signaling Pathway in Ovarian Cancer
Source: Int J Mol Sci. 2025 Jun 19;26(12):5888. doi: 10.3390/ijms26125888 (PMC12193027; doi:10.3390/ijms26125888)
Supplement: Supplementary file 1 [file ijms-26-05888-s001.zip › ijms-3665899-supplementary.pdf]

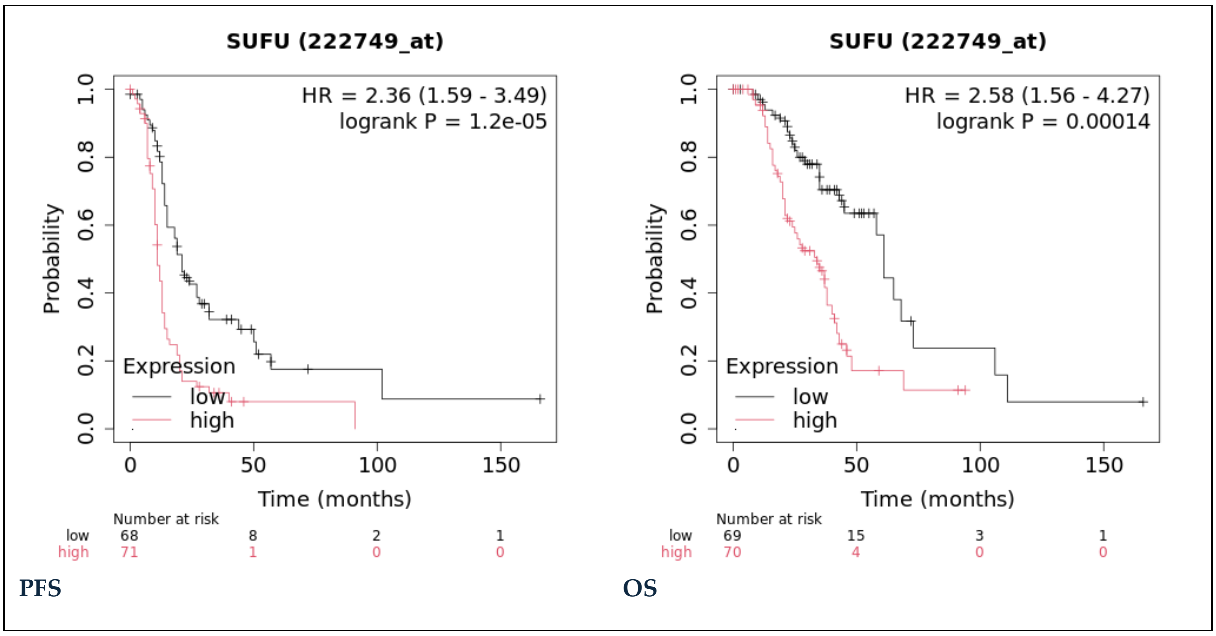

Figure S1.Prognostic significance of *SUFU* expression for progression-free survival (left) and for overall survival (right) in the analysis of the *GSE9891 dataset,* including 139 data of high-grade serous ovarian cancer patients at stages III and IV. HR, 95% CI, and log-rank p-values are given. SUFU: 222749_at; HH: Hedgehog; HR: Hazard ratio. PFS: Progression-free survival, OS: Overall survival
